# Validating Patient-Specific Finite Element Models of Direct Electrocortical Stimulation

**DOI:** 10.3389/fnins.2021.691701

**Published:** 2021-08-02

**Authors:** Chantel M. Charlebois, David J. Caldwell, Sumientra M. Rampersad, Andrew P. Janson, Jeffrey G. Ojemann, Dana H. Brooks, Rob S. MacLeod, Christopher R. Butson, Alan D. Dorval

**Affiliations:** ^1^Department of Biomedical Engineering, University of Utah, Salt Lake City, UT, United States; ^2^Scientific Computing and Imaging (SCI) Institute, University of Utah, Salt Lake City, UT, United States; ^3^Department of Bioengineering, University of Washington, Seattle, WA, United States; ^4^Center for Neurotechnology, University of Washington, Seattle, WA, United States; ^5^Medical Scientist Training Program, University of Washington, Seattle, WA, United States; ^6^Department of Electrical and Computer Engineering, Northeastern University, Boston, MA, United States; ^7^Department of Neurological Surgery, University of Washington, Seattle, WA, United States; ^8^Department of Neurology, Neurosurgery and Psychiatry, University of Utah, Salt Lake City, UT, United States

**Keywords:** direct electrocortical stimulation, electrocorticography, finite element modeling, bioelectricity simulation, patient-specific modeling

## Abstract

Direct electrocortical stimulation (DECS) with electrocorticography electrodes is an established therapy for epilepsy and an emerging application for stroke rehabilitation and brain-computer interfaces. However, the electrophysiological mechanisms that result in a therapeutic effect remain unclear. Patient-specific computational models are promising tools to predict the voltages in the brain and better understand the neural and clinical response to DECS, but the accuracy of such models has not been directly validated in humans. A key hurdle to modeling DECS is accurately locating the electrodes on the cortical surface due to brain shift after electrode implantation. Despite the inherent uncertainty introduced by brain shift, the effects of electrode localization parameters have not been investigated. The goal of this study was to validate patient-specific computational models of DECS against *in vivo* voltage recordings obtained during DECS and quantify the effects of electrode localization parameters on simulated voltages on the cortical surface. We measured intracranial voltages in six epilepsy patients during DECS and investigated the following electrode localization parameters: principal axis, Hermes, and Dykstra electrode projection methods combined with 0, 1, and 2 mm of cerebral spinal fluid (CSF) below the electrodes. Greater CSF depth between the electrode and cortical surface increased model errors and decreased predicted voltage accuracy. The electrode localization parameters that best estimated the recorded voltages across six patients with varying amounts of brain shift were the Hermes projection method and a CSF depth of 0 mm (*r* = 0.92 and linear regression slope = 1.21). These results are the first to quantify the effects of electrode localization parameters with *in vivo* intracranial recordings and may serve as the basis for future studies investigating the neuronal and clinical effects of DECS for epilepsy, stroke, and other emerging closed-loop applications.

## Introduction

Direct electrocortical stimulation (DECS) is used for many clinical, therapeutic, and research applications: mapping eloquent cortex before resection surgery (Berger et al., 1989; Ojemann et al., 1989; Berger and Ojemann, 1992), treating neurological disorders such as epilepsy (Davis et al., 1983; Elisevich et al., 2006; Velasco et al., 2009; Child et al., 2014; Bergey et al., 2015; Lundstrom et al., 2016), promoting rehabilitation after stroke (Brown et al., 2006; Huang et al., 2008; Levy et al., 2008, 2016), and incorporating sensory feedback into brain-computer interfaces (Suminski et al., 2010; O’Doherty et al., 2011; Klaes et al., 2014; Dadarlat et al., 2015; Cronin et al., 2016; Caldwell et al., 2019). All these applications require targeted electrical stimulation of specific anatomical brain regions; however, the mechanisms of DECS that result in a therapeutic effect such as seizure arrest remain unclear. Thus, it is a complex and challenging task to select DECS parameters (active contacts, frequency, pulse width, amplitude, and polarity) that provide optimal clinical benefit.

Computational models of DECS that simulate the voltage generated in the brain are promising tools to understand the electrophysiological response to DECS and optimize stimulation parameters (Guler et al., 2018). The predicted neural response to DECS is dependent on the modeled voltages in the brain; thus, it is essential to understand the effect of modeling parameters on the predicted voltages in the brain. DECS computational models typically use finite element methods (FEM) to represent the three-dimensional geometries of the intracranial tissue and implanted electrodes, while simultaneously incorporating assumed electrical properties of the electrode-tissue interface. Earlier iterations of computational models of DECS used a partial model of the brain, or extruded slab model, to represent a single gyrus and the neighboring sulci to simulate the voltage in motor cortex and the resulting neural activation (Manola et al., 2005, 2007; Wongsarnpigoon and Grill, 2008, 2012; Kim et al., 2011, 2014). These simplified models revealed that the width of the gyrus strongly influences the neural activation distributions below the electrode (Wongsarnpigoon and Grill, 2008). Additionally, these studies found that the electrode polarity and location relative to the gyrus or sulcus activate distinct neuronal populations. These findings highlight the sensitivity to geometric parameters and necessitate the need for models that incorporate patient-specific geometries.

Recent advances in clinical imaging and computing power now enable patient-specific models with geometries extracted from their neuroanatomical imaging data. Several groups have used patient-specific computational models to study neuronal responses in motor cortex (Kim et al., 2012, 2014; Seo et al., 2015, 2016; Fiocchi et al., 2018). However, the predicted voltages have not been validated in humans, presumably due to the challenges of acquiring invasive intracranial voltage recordings during DECS.

Modeling DECS from subdural electrocorticography (ECoG) electrodes introduces unique technical challenges that may have limited the more widespread use of these models. One such challenge is localizing the ECoG electrodes in pre-operative magnetic resonance imaging (MRI) anatomical space using post-operative imaging, typically from computed tomography (CT). During ECoG monitoring, electrodes are placed directly on the cortex to record the underlying electrical activity. In the case of invasive epilepsy monitoring, these recordings help identify the brain regions where seizures occur and whether these regions can be safely resected. Knowing the electrode location in relation to cortical anatomy is imperative to interpret the recorded voltages accurately.

Two factors that contribute to electrode location uncertainty are (1) post-implantation brain shift, and (2) the unknown depth of the cerebral spinal fluid (CSF) between the electrode and the cortical surface. The first of these, brain shift, stems from various causes: brain swelling, brain movement due to the addition of electrodes, CSF drainage, and deformation due to gravity (Hastreiter et al., 2004). The magnitude of brain shift varies on a patient-specific basis and can be as substantial as one or more centimeters (Hill et al., 2000; Dalal et al., 2008). Multiple electrode localization methods address brain shift by projecting the electrodes from their CT post-implant positions to the cortical surface in pre-operative space (Grzeszczuk et al., 1992; Winkler et al., 2000; Morris et al., 2004; Hunter et al., 2005; Sebastiano et al., 2006; Tao et al., 2009; Hermes et al., 2010; LaViolette et al., 2011; Dykstra et al., 2012; Brang et al., 2016). The second factor contributing to uncertainty in electrode location is the depth of the CSF between the electrode and brain tissue, which cannot be determined from postoperative clinical imaging due to the metal artifact around the electrodes and limited image resolution. Furthermore, the CSF depth beneath each electrode may change over time as the brain shifts (LaViolette et al., 2011). CSF is highly conductive compared to brain tissue, and computational studies have shown that the CSF depth affects the current distribution in the brain from DECS (Manola et al., 2005; Wongsarnpigoon and Grill, 2008). However, there are substantial gaps in understanding the consequences of different electrode projection methods and unknown CSF depths on patient-specific DECS model accuracy.

The objective of this work was to validate patient-specific FEM models of DECS that account for brain shift and test model accuracy as a function of CSF depth between the electrode and brain tissue. We modeled the voltages within the brain during DECS for six epilepsy patients with varying levels of brain shift and explored the effects of three established electrode projection methods and three CSF depths on the predicted voltages. We validated these model predictions against clinically recorded voltages measured *in vivo* during DECS and identified the model parameters that predicted the most accurate voltages. These findings will empower future studies with accurate and robust models to investigate the underlying mechanisms of DECS therapy for epilepsy, stroke rehabilitation, and brain-machine interface applications.

## Materials and Methods

### Participants

Six patients with intractable epilepsy underwent acute clinical monitoring with implanted ECoG electrodes at Harborview Medical Center (Seattle, WA, United States) for consideration of surgical resection of epileptogenic tissue. The ECoG grids and strips had an exposed electrode diameter of 2.3 mm and an inter-electrode distance of 1 cm center-to-center (Ad-tech Medical, Racine, WI, United States). They were implanted at locations determined by the clinical team to be most likely to identify seizure foci. All patients provided informed consent for the protocol approved by the University of Washington Institutional Review Board.

### Stimulation and Recording

**FIGURE 1:**
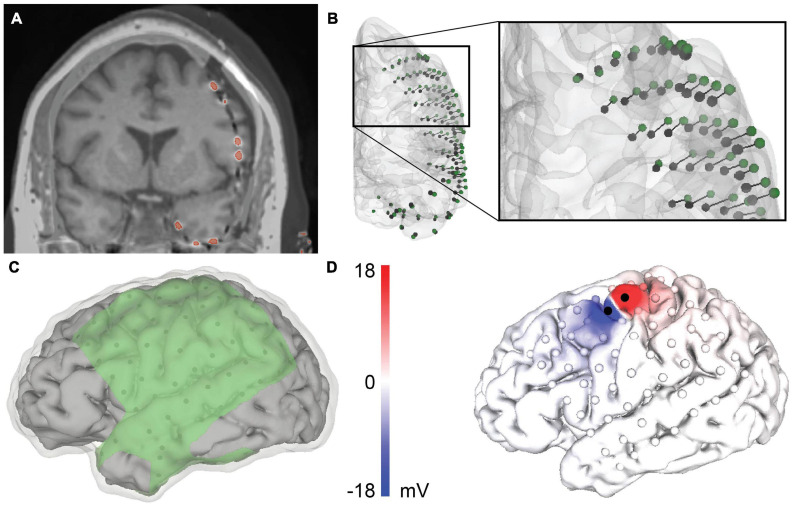
Patient-specific modeling pipeline for a representative patient, patient 4. **(A)** Merged co-registered magnetic resonance imaging (MRI) and computed tomography (CT). The thresholded electrodes from the CT are shown in red. Due to brain shift, some electrodes appear to be inside the brain and require projection to the closed gray matter surface. **(B)** Anterior view of the rigid electrode centroids (black) with a vector to the projected Hermes localization (green). **(C)** The geometry of the volumetric mesh: cerebral spinal fluid (CSF) (transparent), gray matter (gray), and insulating sheet (green). The electrodes are shown in black on the underside of the insulating sheet. **(D)** The predicted voltage on the cortical surface during bipolar stimulation between the black electrodes.

Biphasic, bipolar current-controlled stimulation was delivered across two neighboring electrodes on the ECoG grid (Figure 1D) with a pulse width of 1.2 ms. Table 1 outlines the electrode pair, stimulation amplitude, and number of stimulation pulses delivered for each patient. The 62 passive (i.e., non-stimulating) grid electrodes were recorded at a sampling rate of 12,207 Hz. We baseline-corrected each stimulation pulse to the mean pre-stimulus signal from 50 to 5 ms before stimulation onset.

**TABLE 1:** Stimulation parameters applied in the cohort.

**Patient**	**Stimulation amplitude (mA)**	Electrodes stimulated (−/+)*	**Number of stimulation pulses**
1	3.50	B4/B3	10,005
2	1.75	A1/B1	10,000
3	2.50	A3/B3	3,014
4	0.75	B5/B6	10,000
5	1.75	C6/D6	3,001
6	0.75	A6/A5	7,014

*See Figure 5B for visualization of grid coordinates.

**FIGURE 2:**
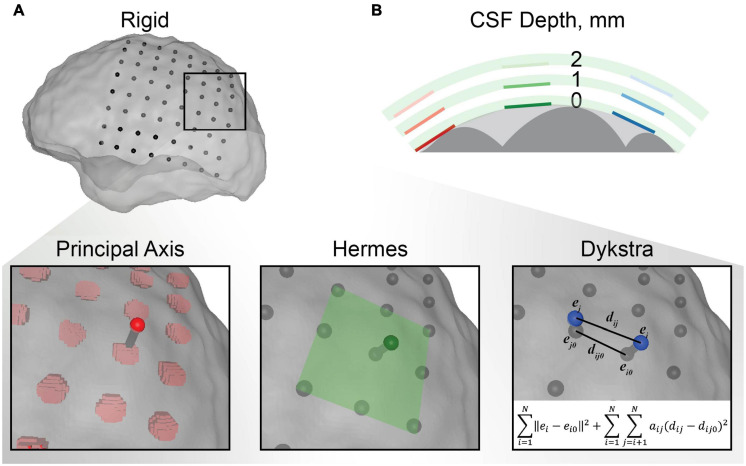
Electrode localization methodology. **(A)** An example of each electrode projection method for a single electrode in the electrocorticography (ECoG) grid. The centroids for the rigid electrode localizations from the CT are shown in black with respect to the transparent closed gray matter surface used as the final destination for projection. The principal axis method independently projected each electrode centroid along the principal axis of the electrode artifact from the CT. The Hermes method projected each electrode in the direction normal to the plane defined by the electrode and its nearest neighbors. The Dykstra method implemented the shown constrained energy-minimization algorithm. This algorithm minimized both electrode displacement (∥e_i_−e_i0_∥^2^) and inter-electrode distance deformations (*a_ij_*(*d_ij_*−*d_ij0_*)^2^) where *a_ij_* = 1 for neighboring electrodes and 0 for distant electrodes. **(B)** Schematic representation of the CSF depth below the electrode. Each electrode was modeled with 0, 1, and 2 mm of CSF between the electrode and closed gray matter surface (the surface boundary of the light gray area) for each electrode projection method. The gray matter is shown in dark gray and the insulating grid is shown in green for each CSF depth.

**FIGURE 3:**
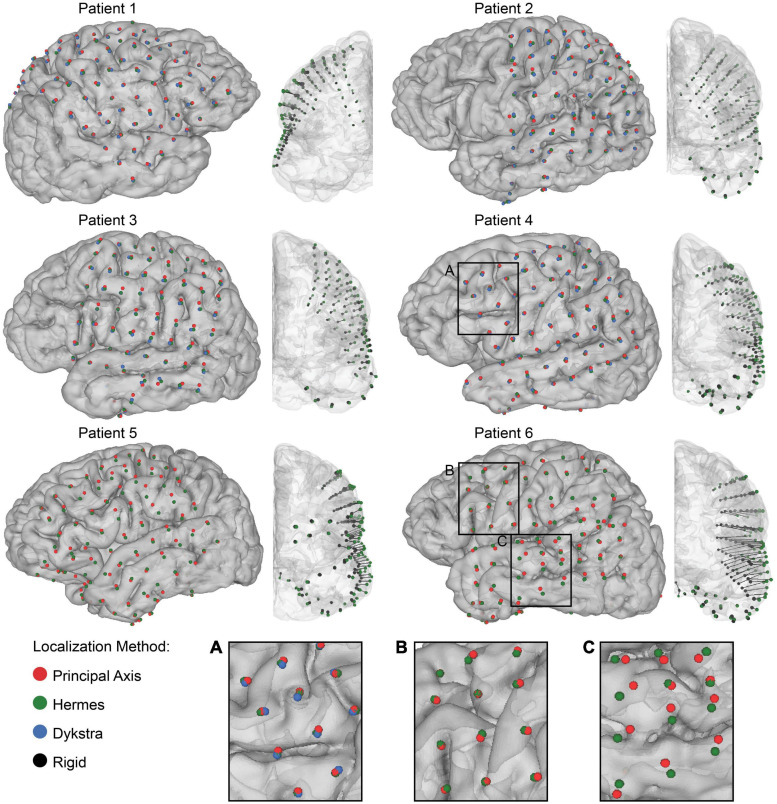
Electrode localization and quantification of brain shift. Final electrode localization for all projection methods for 0 mm CSF depth in each patient. The centroids indicate the final electrode localization on the patient-specific gray matter surface: principal axis (red), Hermes (green), and Dykstra (blue) (*left*) and the anterior view of the rigid electrode localization from the CT (black) with a vector to the projected Hermes localization on the gray matter surface (*right*). Both strip and grid ECoG electrodes are shown. Note that the Dykstra method did not converge for patients 5 and 6. Across projection methods, the centroid locations were similar for many of the patients **(A)**, but varied in others by converging **(B)**, and diverging **(C)** in separate brain regions.

**FIGURE 4:**
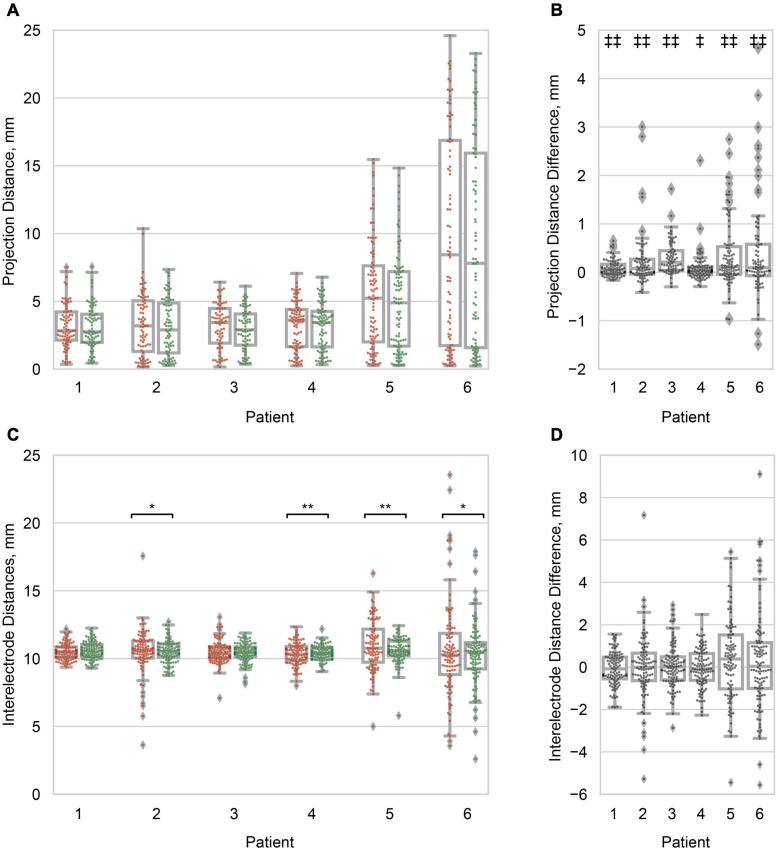
Projection and inter-electrode distances. **(A)** Boxplots of the principal axis (red/*left*) and Hermes (green/*right*) projection distance for all electrodes within a patient. Distribution variances were not significantly different between projection methods (α = 0.05). Patients are ordered from least to greatest median Hermes projection distance. **(B)** Boxplots of the pairwise projection distance difference: principal axis–Hermes. In all patients the projection distance was significantly smaller for the Hermes method. **(C)** Boxplots of the inter-electrode distances for the principal axis (red/*left*) and Hermes (green/*right*) projection methods. Each data point represents a pair of neighboring electrodes on the ECoG grid. The inter-electrode variance was statistically less for the Hermes method for patients 2, 4, 5, and 6. **(D)** Paired inter-electrode distance differences (principal axis–Hermes) were not statistically significant (α = 0.05). For **(A,C)**, significantly different variances between the principal axis and Hermes methods are denoted with *p < 0.01; **p < 0.001. For **(B,D)**, significantly different paired distances are denoted with ^‡^*p* < 0.01; ^‡⁣‡^*p* < 0.001. All distances are for the 0 mm CSF models.

**FIGURE 5:**
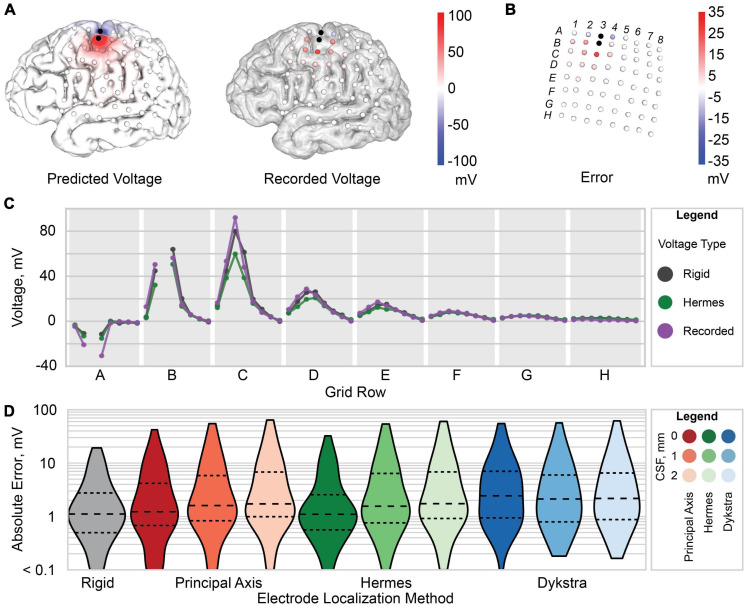
Voltage and error for patient 3. **(A)** Predicted voltage on the gray matter surface (*left*) and recorded voltage at each recording electrode (*right*). **(B)** Raw error (predicted voltage subtracted from the recorded voltage) at each electrode. **(C)** Predicted voltage for the Hermes method with 0 mm CSF (green), rigid model (gray), and recorded voltage (purple). Each gray region marked with a letter represents a row of electrodes on the ECoG grid [labels shown in panel **(B)**]. **(D)** Violin plot of the absolute error between the predicted and recorded voltages for each electrode localization method: rigid (gray), principal axis (red), Hermes (green), and Dykstra (blue) with 0, 1, and 2 mm of CSF from left to right for each method. The dashed line is the median, and smaller dashed lines demark the interquartile range.

We extracted a single voltage at each electrode to compare against the predicted volume conduction voltages with an automated algorithm that identified the quasi-static interval of each stimulation pulse. We calculated the mean over all the recorded stimulation pulses for each channel, and found the differentiated averages as the first difference of those means. We converted each sample in the differentiated averages to their z-score equivalents: near zero during the middle of each phase, and substantially non-zero near phase onsets and offsets. We used a threshold of ±1.5 on the z-scored differentiated averages to determine the start and end of each pulse phase. We determined the average steady-state voltage between onset and offset, shortened by three samples in each direction to ensure that our extracted voltages were within the quasi-static interval of the stimulation pulse. This signal extraction algorithm has been previously validated with a saline phantom using our recording setup (Caldwell, 2019).

### Patient-Specific Models

We built nine whole-brain finite element meshes per patient to assess the effects of electrode projection method and CSF depth on predicted voltages. The different models incorporated three common electrode projection methods to account for brain shift: principal axis (Brang et al., 2016), Hermes (Hermes et al., 2010), and Dykstra (Dykstra et al., 2012). We combined each projection method with three depths of the CSF from the electrode to the cortical surfaces. The CSF depth between the dura and cortical surface varies across the brain and across patients and has been reported as 2–3 mm (Wagner et al., 2004). The thickness of the ECoG grid, 0.5 mm, presumably reduces this depth. We therefore modeled CSF depths of 0, 1, and 2 mm.

#### Image Registration and Tissue Segmentation

We rigidly co-registered the postoperative CT to the pre-operative T1-weighted structural MRI using BRAINSFit rigid registration (Johnson et al., 2007) implemented in 3D Slicer^1^ (Fedorov et al., 2012; Figure 1A). We segmented the T1-weighted image and created surface meshes–of the gray matter, white matter, and ventricles–with the command line tool mri2mesh in the SimNIBS software package (Thielscher et al., 2015). We then checked the automated segmentations for accuracy against the MRI and manually made necessary segmentation edits in Seg3D^2^. If edits were made, a second run of mri2msh incorporated those edits into the surface meshes. We generated a closed gray matter surface onto which we could project the electrodes with 3DSlicer’s closing filter. This filter performed a morphological dilation-then-erosion to the volumetric gray matter segmentation output from mri2mesh. This segmentation filled in the sulci, ensuring the ECoG electrodes would be projected onto a closed cortical surface and not into a sulcus. The resulting closed gray matter segmentation was then dilated to produce the CSF segmentation, using the 3DSlicer Segment Editor module. We then exported the closed gray matter and CSF segmentations as triangular surfaces for volumetric meshing.

#### Electrode Localization

After co-registering the pre- and post-operative images, some electrodes appeared to be inside the brain, making it necessary to account for brain shift by projecting the electrodes to the closed gray matter surface (Figures 1A,B). The electrodes were localized by first calculating the “rigid” centroid of each electrode from the thresholded electrode artifact in the CT. Each centroid was projected to the closed gray matter surface using three electrode projection methods–principal axis (Brang et al., 2016), Hermes (Hermes et al., 2010), and Dykstra (Dykstra et al., 2012; Figure 2)–to contrast their effects on the simulated voltages in the brain. Briefly, the principal axis method calculates each electrode’s longitudinal axis from its artifact and projects each electrode independently along its respective principal axis. The Hermes method projects each grid electrode in the direction normal to the plane constituting the electrode and its nearest neighbors. The Dykstra method implements a constrained energy-minimization algorithm that minimizes both electrode displacement and inter-electrode distance deformations. For each method, resulting electrode locations were used for the models with a CSF depth of 0 mm. We modeled each electrode as a 2.3 mm diameter disk of 73 nodes, oriented parallel to the nearest triangular element of the closed gray matter surface. We then moved each electrode 1 or 2 mm along the normal vector of the nearest triangle surface for models with a CSF depth of 1 and 2 mm, respectively. All projection methods were implemented with MATLAB and Python.

#### Head Model and Simulations

Our meshing approach incorporated surface meshes that segmented each region into the final volumetric mesh. This approach advantageously maintains clean boundaries between regions but necessitates nested surfaces. We used the gray matter, white matter, and ventricle surfaces from the mri2mesh output and the CSF surface from “Image Registration and Tissue Segmentation.” We then created an ECoG grid/strip surface mesh for each projection method and CSF depth. Our major challenge was constructing the insulating sheet around the projected electrodes while following the contours of the closed gray matter surface. We triangulated the nodes of the projected electrodes and mapped the triangulated surface onto the closed gray matter surface. We extruded the resulting closed gray matter patch 0.5 mm to create a three-dimensional surface mesh. The final insulating sheet mesh for each electrode localization followed the contours of the closed gray matter surface and incorporated the projected 2.3 mm diameter electrodes (Figure 1C). We then generated tetrahedral finite element meshes for each projection method and CSF depth in SCIRun 5.0^3^ with the InterfaceWithTetGen module (Si, 2015). The resulting volumetric meshes had approximately 0.9 million nodes and 4.9 million elements per mesh. Isotropic conductivities taken from the literature were used for each tissue and electrode compartment (Table 2). To simulate the bipolar stimulation applied during the clinical session, we calculated the voltage at each node in the tetrahedral mesh using the Poisson equation, ∇⋅σ∇*V*_*e*_ = −*i* for *x* in Ω_*C*_, where Ω_*C*_ is the volumetric mesh, and electrode current source *i* = *I*_0_δ*x*_0_ of stimulation amplitude *I_0_* as reported in Table 1. We used Neumann boundary conditions, ∂_Γ_V*_e_* = 0 for *x* in *Γ*_*Neu*_, where *Γ*_*Neu*_ is the mesh boundary. The system of equations was solved with SCIRun 5.0 using the conjugate gradient solver, Jacobi preconditioner, and an error tolerance of 1 × 10^–8^. A representative solution is shown in Figure 1D. We repeated this process for all nine meshes for each of the six patients.

**TABLE 2:** Head model conductivities of each compartment.

**Compartment**	**Conductivity (S/m)**	**References**
Gray matter	0.330	Haueisen et al., 1997
White matter	0.142	Haueisen et al., 1997
Ventricles	1.790	Baumann et al., 1997
CSF	1.790	Baumann et al., 1997
Silicone insulating sheet	1 × 10^–10^	Wei and Grill, 2005

### Rigid Model

Volume conduction and, therefore, the recorded voltages are highly influenced by the stimulation and recording electrodes’ relative spacing, which we manipulate when we project the electrodes onto the closed gray matter surface. To evaluate the effects of electrode spacing, we additionally built a “rigid” head model with the original electrode locations in CT space. The rigid model preserves the physical inter-electrode distances of the ECoG grid. However, it sacrifices the accuracy of the gray matter-CSF interface, which divides regions with a ∼five-fold difference in conductivities. After co-registration to the pre-operative MRI, some rigid electrode locations appear to be inside the brain (Figure 1A). Therefore, we manually edited the gray matter segmentation by removing any tissue voxels above the electrode grids and strips. Thus, the rigid model resulted in a clipped version of the pre-operative brain surfaces covered by the ECoG electrodes. Simulations were performed with the same methods as described in “Head Model and Simulations.”

### Model Validation and Statistical Analysis

To assess the level of brain shift across the ECoG electrodes, we calculated the projection distance, the length of the vector from the rigid electrode centroid to the projected centroid, for each electrode for all projection methods. To determine if the inter-electrode distances were affected by projection, we quantified the inter-electrode distance between neighboring electrodes for all projection methods. The Brown-Forsythe test assessed the equality of variance in projection distances and inter-electrode distances between the principal axis and Hermes projection methods for each patient (α = 0.05) (Brown and Forsythe, 1974). The Wilcoxon signed-rank test assessed the paired difference in projection distance and inter-electrode distance between the principal axis and Hermes projection method for each patient (α = 0.05).

We then considered how different projection methods and CSF thicknesses affected the simulated voltages at the electrodes. We evaluated the absolute error, |V_rec_−V_sim_|, between the simulated voltages(*V*_*sim*_) and recorded voltages (*V*_*rec*_). The Wilcoxon signed-rank test assessed the equality of the voltage absolute error distributions, compared for each projection method at all CSF depths (0, 1, and 2 mm; α = 0.05). The Holm-Bonferroni procedure controlled for multiple comparisons. Next, we performed a linear regression to predict each patient’s recorded voltages based on their simulated voltages. We used the Pearson correlation coefficient to measure the similarity between the two distributions of voltages, and the slope of the regression to evaluate the similarity of the magnitudes of the voltages. Finally, we performed a linear regression across the entire population by normalizing each subject’s experimental data to the voltages that would have been each recorded in response to 1 mA stimulation, presuming linear scaling.

## Results

### Electrode Localization and Quantification of Brain Shift

We first visualized the rigid and projected electrode localizations across projection methods (Figure 3). The number of localized grid and strip electrodes per patient ranged from 72 to 101. The Dykstra projection method did not converge for patients 5 and 6 due to substantial brain shift. Because this method was not reliable across all patients, only the principal axis and Hermes methods were used for further analyses (see Supplemental Figure for additional Dykstra results). We observed similar centroid locations between projection methods for most electrodes (Figure 3). However, for some electrodes, the principal axis and Hermes projection methods yielded noticeably different centroid locations, occasionally even on different gyri (Figure 3C). For some patients, the centroid locations were similar between projection methods across most of the brain (Figure 3, e.g., patient 4). In other patients, the centroid locations from the distinct projection methods converged in some brain regions but diverged in others (Figure 3, e.g., patient 6).

We then quantified the distribution of projection distances at a single CSF depth of 0 mm across electrodes as a metric for brain shift. We observed significantly different distributions of projection distances within patients, where the projection distances for the Hermes method were less than those for the principal axis method for all patients (Figures 4A,B). Differences in the projection distance variances between the principal axis and Hermes projection methods were not statistically significant for any of the patients (Figure 4A). Median projection distances varied across patients; patients 5 (4.90 mm) and 6 (7.80 mm) had a greater median Hermes projection distance than patients 1, 2, 3, and 4 (2.75, 2.89, 2.90, and 3.44 mm, respectively; Figure 4A). Projection distances are shown for the Dykstra method in Supplementary Figure 1A.

We found that projection of the electrodes increased the distances between neighboring electrodes beyond the 10 mm actual value of the physical grid; the range of median inter-electrode distances across patients was 10.27–10.76 mm for the Hermes electrode projection and CSF depth of 0 mm (Figure 4C). We found no significant paired difference in inter-electrode spacing between the principal axis and Hermes methods for any patient (Figure 4D). Although projecting the electrodes resulted in less than a millimeter increase in median inter-electrode distance, individual inter-electrode distances ranged from 2.80 to 19.98 mm across patients (Figure 4C). We observed a larger spread in inter-electrode distances for patients with larger projection distances (i.e., greater brain shift). The Hermes method yielded inter-electrode distances with significantly less variance than the principal axis method for 4 of 6 patients (2, 4, 5, and 6), most notably for the patients with larger projection distances (Figure 4C). Inter-electrode distances are shown for the Dykstra method in Supplementary Figure 1B.

### Simulated Voltages and Voltage Errors

We then evaluated the spatial voltages and error between the simulated and recorded voltages. Results are shown for a Hermes projection with 0 mm CSF for a representative patient, patient 3 (Figures 5A,B). We found that the electrodes (A2, A4, B2, B4, and C3) nearest to the two stimulation electrodes (A3 and B3) had a larger amplitude raw error compared to electrodes farther from the stimulating electrodes (Figures 5B,C). This trend persisted across all patients for all electrode projection methods and CSF depths.

We compared the projected electrode models, which had an increased inter-electrode distance, to the “rigid” model that retained the original inter-electrode spacing of 10 mm but sacrificed accurate cortical anatomy. For patient 3, the simulated voltages for both the rigid and Hermes model closely follow the recorded voltage. However, for larger potentials–i.e., closer to the stimulating electrodes–the Hermes model under predicts the voltage (Figure 5C). For clarity, Figure 5C shows only the voltages predicted with the Hermes method; a complete summary for principal axis and Dykstra methods is shown in Supplementary Figure 2A. We calculated the absolute error across CSF depths to determine which CSF depth predicted more accurate voltages. Figure 5D shows the data for a representative patient, patient 3. We found the median absolute error increased with increasing CSF depth for both the principal axis and Hermes methods (Figure 5D). Supplementary Figure 2B includes voltages predicted by the Hermes projection method at each electrode for all three CSF depths. In summary, models of patient 3 that included 0 mm of CSF between the electrodes and cortical surfaces yielded the most accurate simulations.

**FIGURE 6:**
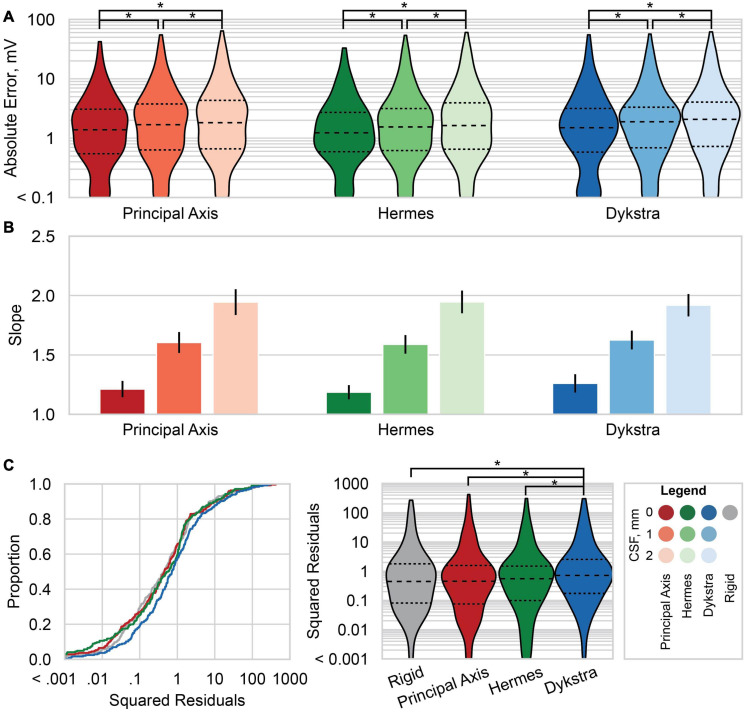
Absolute errors and linear regression results across patients. **(A)** Violin plots of the absolute error between the predicted and recorded voltages for each electrode localization method: principal axis (red), Hermes (green), and Dykstra (blue) at–from left to right within each method–0, 1, and 2 mm of CSF. Results for the principal axis and Hermes methods summarize all six patients; results for Dykstra comprise only the four patients for whom that method converged. Dashed line is the median, and smaller dashed lines bound the interquartile range. **(B)** The slope of the linear regression of the predicted and recorded voltages at a stimulation amplitude of 1 mA across all patients for each electrode localization method and CSF depth. The linear regressions are across six patients for the principal axis and Hermes methods, and across four patients for the Dykstra method. The error bars show the 95% confidence interval of the slope. Slopes closer to one better predict the magnitudes of the recorded voltages. **(C)** The squared residuals of the linear regression of the predicted and recorded voltages at a stimulation amplitude of 1 mA across the four patients for whom all electrode localization methods worked (patients 1–4). Shown are both the empirical cumulative distribution function of the squared residuals (*left*) and violin plots of the squared residuals (*right*). Significant pairwise comparisons between **(A)** CSF levels within a projection method and **(C)** between electrode projection methods after correction for multiple comparisons are shown with *p < 10^– 4^.

We next compared the absolute error at different CSF depths across patients: the median absolute error increased with increasing CSF depth for all three projection methods (Figure 6A). The absolute error distributions were significantly different for all pairwise comparisons after correction for multiple comparisons.

### Linear Regressions

We then examined the linear regressions across all patients for the three-electrode projection methods and CSF depths. The slopes of the linear regressions are shown in Figure 6B; slopes closer to one better predict the magnitudes of the recorded voltages. For all three electrode projection methods, the CSF depth with the slope closest to one, and therefore the depth that resulted in the best fit to the data was 0 mm. The slope increased with CSF depth for all electrode projection methods. We note that the Dykstra regressions are across the four patients where the method converged whereas the principal axis and Hermes methods are across all six patients. Based on the results showing that models with a CSF depth of 0 mm have less absolute error and better predict the magnitude of the recorded voltages (slopes closer to one), moving forward we present data only for simulations with 0 mm CSF.

We then examined the squared residuals of the linear regressions for electrode localization methods. We show the cumulative distribution function (CDF) of the squared residuals for each electrode localization method on the left of Figure 6C. The squared residuals were significantly greater for the Dykstra method compared to all other methods (Figure 6C-right; α = 0.05, Holms-corrected pairwise comparisons).

**FIGURE 7:**
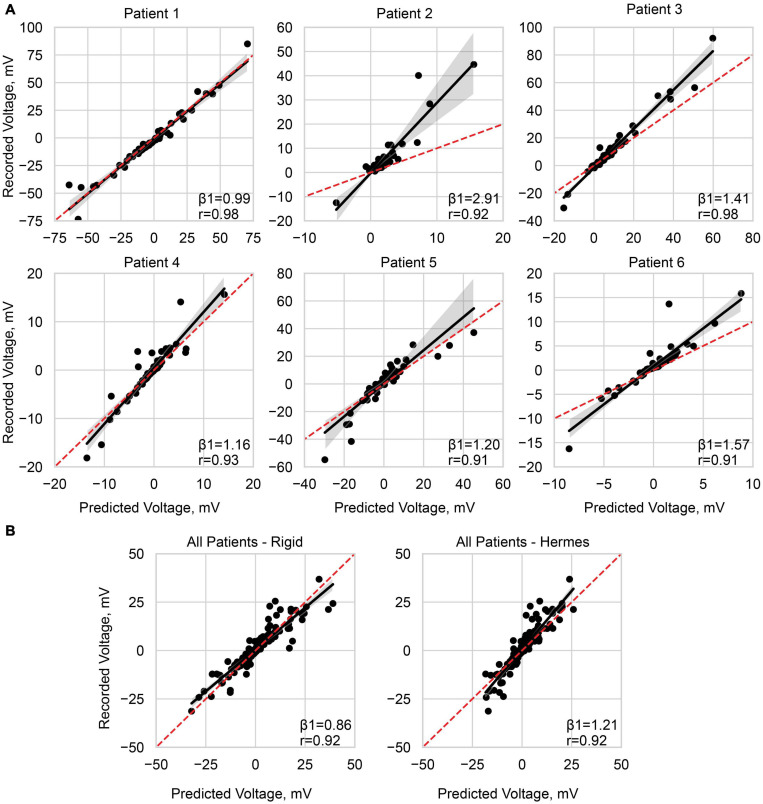
Predicting the recorded voltage. **(A)** Linear regression of the predicted voltage using the Hermes projection method and the clinically recorded voltages for each patient. **(B)** Linear regression across all patients, with a normalized stimulation amplitude of 1 mA, for the rigid method and Hermes method. Each data point represents a single electrode on the ECoG grid. The red dashed line is unity, and the shaded gray region is the 95% confidence interval. Slope (β1) and Pearson correlation coefficient (*r*) are shown on each plot, and additional statistics are available in Table 3.

We report slopes of 1.25, 1.21, and 1.26 for the principal axis, Hermes, and Dykstra electrode projection methods, respectively, at a depth of 0 mm CSF across patients (Figure 6B). The electrode projection method that resulted in the best patient-specific linear fit was Hermes in 4/6 of the patients (patients 1, 3, 5, and 6), principal axis for 1/6 patients (patient 4), and Dykstra for 1/6 patients (patient 2). Because the Hermes method best predicted the magnitudes of the recorded voltages for the majority of patients, we show the patient-specific linear fits and correlation values for the Hermes projection method in Figure 7A, with additional regression statistics in Table 3. For the Hermes projection method, the mean correlation was 0.94 ± 0.03 (SD) and the mean slope was 1.53 ± 0.64 (SD) across all six patients. Simulated voltages nearly matched recorded voltages for 5/6 patients. For patient 2, however, the recorded voltages were 2.9× greater than those predicted by simulation.

**TABLE 3:** Predicting the recorded voltage: linear regression statistics.

**Patient**	**Slope (β1)**	**Standard Error of β1**	***t***	***p(t)***	***R*^2^**	***F***	***p(F)***
**Hermes, 0 mm CSF**							
1	0.99	0.03	36.24	<0.001	0.96	(1,60) = 1313	<0.001
2	2.91	0.16	18.31	<0.001	0.85	(1,60) = 335	<0.001
3	1.41	0.03	42.05	<0.001	0.97	(1,60) = 1769	<0.001
4	1.16	0.06	18.99	<0.001	0.86	(1,59) = 361	<0.001
5	1.20	0.07	16.56	<0.001	0.82	(1,60) = 274	<0.001
6	1.57	0.09	17.54	<0.001	0.84	(1,60) = 308	<0.001
All patients	1.21	0.03	44.64	<0.001	0.84	(1,368) = 1992	<0.001
**Rigid**							
All patients	0.86	0.02	45.36	<0.001	0.85	(1,368) = 2057	<0.001

We then performed a correlation and linear regression analysis across all patients for a stimulation amplitude of 1 mA for all electrode localization methods. We show the results for the Hermes and rigid methods in Figure 7B. The correlation coefficients were 0.92 for both the rigid and Hermes models, with slopes of 0.86 and 1.21, respectively. The linear regression statistics are shown in Table 3.

## Discussion

In this study, we validated patient-specific FEM models of DECS with recorded voltages in humans. We determined the modeling parameters that best predicted the recorded voltages across six patients were a CSF depth of 0 mm and the Hermes projection method. Three main results serve as the basis for this determination. First, models with 0 mm of CSF between the electrode and closed gray matter surface resulted in the least absolute error between the simulated and recorded voltages for 5/6 patients. Second, the Hermes projection method better-preserved inter-electrode distances (i.e., closer to 10 mm) than the principal axis method (Figure 4C). Third, the Hermes method had a lower median absolute voltage error than the principal axis and Dykstra methods across patients (Figure 6A). We further validated this model across patients with a linear regression predicting the recorded voltages based on the simulated voltages and found a strong linear relationship (correlation of *r* = 0.92 and β1 = 1.21). Together, these results support using the Hermes projection method to account for brain shift, and that using 0 mm of CSF below the electrode results in more accurate voltage estimation.

### Electrode Localization Uncertainty

Failing to project electrodes to account for post-implantation brain shift often results in obviously erroneous electrode locations, including within the brain; to rectify this situation, electrodes can be projected to the closed gray matter surface. Although validating the projected electrode locations was outside the scope of this study, previous studies have validated these projection methods with a combination of known electrode geometries and intra-operative photos. A study validating the principal axis method reported a mean error of 0.4 mm across 10 patients (Brang et al., 2016), while a different study validating the Hermes method reported a median error of 2.4 mm across six patients (Hermes et al., 2010). The errors reported in these validation studies are on the order of a single electrode diameter (2.3 mm), making it likely that the projected electrodes result in localization to the true anatomical landmark (gyrus or sulcus) necessary for accurate interpretation of ECoG recordings.

However, we note that different methods occasionally projected the same electrode onto two distinct gyri, particularly for patients with greater projection distances, i.e., patients 5 and 6 (Figure 3). Features of each projection method underlie the differences in projection location. The principal axis method occasionally resulted in noisy projection vectors seemingly because each electrode is projected independently based on its surrounding artifact in the CT image. Therefore, poor imaging quality or resolution may introduce more uncertainty in the projection vector. This sensitivity may explain the significantly larger projection distances for the principal axis method compared to the Hermes method for all patients (Figure 4B). A projection vector that is slightly off could result in an increased distance to the closed gray matter surface. In contrast to the principal axis method, the Hermes and Dykstra methods incorporate information from neighboring electrodes, making spurious projection vectors for a single electrode less likely. We do not recommend the Dykstra projection method as it did not converge for patients 5 and 6. This nonconvergence likely stems from the substantial brain shift observed for these patients. For the other projection methods, the median and range of the projection distances were greater for these patients compared to the other four patients (Figure 4A). The constrained energy-minimization Dykstra algorithm may have failed to converge because such large projection distances were necessary across a majority of the electrodes.

For all projection methods, intraoperative imaging could help validate electrode localization errors (Pieters et al., 2013). However, ECoG electrodes may slide an average of 4.0 mm relative to the cortical surface throughout acute monitoring (LaViolette et al., 2011). It is unknown if CT imaging throughout the monitoring period, instead of directly after implantation as was done in our study, would significantly influence projected electrode locations. This information would be valuable to understanding the dynamics of brain shift, but it may not justify the additional radiation exposure from CT imaging. Combined with evidence from the aforementioned validation studies, our results support that our electrode localizations are accurate to within the limits of existing clinical imaging and enable interpreting ECoG recordings within anatomical landmarks (Hermes et al., 2010; Brang et al., 2016). Until we better understand how ECoG electrodes shift throughout the course of implantation, applying caution when interpreting ECoG recordings with inherent uncertainty in the true location of the electrode is warranted. This uncertainty in electrode location also applies to chronic ECoG electrodes implanted for closed-loop applications and may influence the optimal time to acquire post-operative imaging for patient-specific FEM models for these applications.

### Influence of Projection on Inter-Electrode Spacing and Model Accuracy

Although electrode locations enable accurate ECoG data interpretation, the effects of electrode projection on inter-electrode spacing were previously unknown. Volume conduction is dependent on the relative distance between stimulating and recording electrodes; thus, the best projection methods would maintain both inter-electrode spacings and accurate neuroanatomical locations. To understand the effects of altering the inter-electrode spacing, we created the rigid model, which retains an ECoG grid’s correct geometry. This approach requires altering the brain geometry. Therefore, the rigid method sacrifices correct anatomical information. Without an accurate spatial interpretation of brain activity, our computational model predictions have no context for therapeutic interpretations that require precise targeting to specific anatomical regions.

Knowing that the inter-electrode spacing is important for accurate volume conduction modeling, we wanted to minimize the change in inter-electrode spacing after electrode projection. We found that the inter-electrode distances between the Hermes and principal axis method were not significantly different, but the Hermes method had a significantly smaller inter-electrode variance in 4/6 patients. The Hermes method’s smaller variance retained inter-electrode distances closer to the median values–ranging from 10.27 to 10.76 mm–across all electrodes, thus outperforming the principal axis method (Figure 4C). These results are likely because the Hermes method had significantly smaller projection distances than the principal axis method, allowing the inter-electrode distances to remain more tightly bound around the rigid distance of 10 mm. Additionally, neighboring electrodes’ projection vectors were likely more similar between neighboring electrodes because these electrodes defined the plane normal to the projection vector, whereas the principal axis projection vector was treated independently for each electrode.

We additionally considered the effects of increased inter-electrode distances on voltage predictions by comparing the Hermes model to the rigid model. Our regression results across all patients suggest that the rigid model, which by design preserves precise inter-electrode spacings, overestimates recorded voltages with a slope of 0.86 (Figure 7B). In contrast, the Hermes model underestimated the same voltages with a slope of 1.21. The rigid model overestimates may be due to decreased CSF in the sulci due to the closed gray matter surface, whereas CSF is present in the sulci of the 0 mm CSF models. The differences in these models’ results do not support any direct conclusions about the influence of altered inter-electrode spacing on estimated voltages in the brain. However, we would like to highlight the importance of accurate neuroanatomical locations in the context of therapeutic applications over maintaining the inter-electrode spacing: i.e., accurately estimating the voltage at a recoding electrode is moot if that electrode is modeled at the incorrect brain location.

### DECS Model Validation

Computational models are powerful tools to quickly and cost-effectively predict electrophysiological and clinical responses to DECS; however, there has been limited validation of their application in humans. Phantom models are often used to validate measurements that are challenging to obtain in humans. Previous work by Kim et al. (2015) used an Agar/NaCl phantom based on a human MRI to record voltages during DECS and an FEM model to predict the phantom voltages. They found an average relative difference between predicted and measured voltages throughout the brain of 10.3%. However, the use of a phantom model eliminated the need to address post-implantation brain shift.

To better understand the effects of conductivity selection and automated segmentation pipelines, electric field models have been validated against *in vivo* measurements from intracranial contacts during transcranial electrical stimulation (Huang et al., 2017; Puonti et al., 2020). These studies co-registered pre- and post-operative MRI to localize electrodes; they accounted for brain shift by back-projecting the electrodes onto the pre-operative cortical surface (Yang et al., 2012). The predicted electric fields correlated well to the cortically measured values with a correlation coefficient of *r* = 0.86 (Huang et al., 2017).

Our validation builds on the previous work by incorporating *in vivo* data from six patients, quantifying the effects of electrode projection to account for brain shift, and investigating the influence of CSF depth on predicted voltages. We were able to accurately predict DECS voltages across six patients with variable amounts of brain shift (slope of 1.21 and correlation coefficient *r* = 0.92). Interestingly, the models with a CSF depth of 0 mm best predicted the recorded voltages. As CSF depth increased, the slope of the linear regressions also increased. This dependence suggests that the amount of CSF between the electrode and cortical surface is closer to 0 than 1 mm even after several days of implantation and brain shift. The observed sensitivity to CSF is consistent with previous work demonstrating how the CSF depth or segmentation has a large effect on the current distribution in the brain. Shunting effects likely underlie this sensitivity, which highlights the importance of accurate CSF depths in future models of DECS (Manola et al., 2005; Wongsarnpigoon and Grill, 2008; Puonti et al., 2020). Enforcing a uniform CSF depth over the closed gray matter surface limits the accuracy of this approach, because that depth may vary spatially and with head position (Rice et al., 2013). However, without novel imaging revealing how CSF depth varies with location, implementing a uniform CSF depth minimizes the free parameters in the model, and a depth of 0 mm yields the most accurate results.

### Model Limitations

Although our predictions strongly correlated with the recorded voltages, suggesting accurate voltage distributions, addressing other modeling parameters may improve results. For example, the Hermes model accurately predicted the recorded voltages for 5/6 patients. However, the linear regression slope for patient 2 was far from unity, greatly underestimating the recorded voltages (slope = 2.91; Figure 7A). We observed that the stimulation electrodes for this patient were localized over a sulcus after the projection. As an ad hoc analysis, we created an additional Hermes model with a closed gray matter surface; removing the sulci from the model ensured that the electrodes were localized over brain tissue. This ad hoc model decreased the slope for all patients. The decrease in slope greatly improved the fit for patient 2 (slope = 1.28) while worsening the slopes for patients previously close to unity. We hypothesize that recordings from contacts near a sulcus are more sensitive to geometric errors, including incorrect projection locations and inaccurate representations of the sulcal widths. It is worth noting that the gray matter surfaces in our standard models overestimate the sulcal width. The larger sulci enable cortical surface models with no overlapping edges: a necessary constraint to create the volumetric finite element meshes. Based on extruded slab models of epidural cortical stimulation, we know that the neurons activated within a gyrus depend on the gyrus width (Wongsarnpigoon and Grill, 2008). Future work could explore the influence of electrode localization errors and inaccurate sulcal widths by systematically ranging gyri inflation or stepping each electrode through space and orientation. We recommend additional studies to better understand the influence of gyrus thickness and incorrect electrode localization on predicted voltages in the brain.

Another parameter that may improve the accuracy of patient-specific DECS models is the choice of tissue conductivities. In the aforementioned transcranial stimulation validation study, Huang et al. (2017) optimized the patient-specific isotropic conductivities to minimize the error of the electric field. The optimized conductivities produced significantly better correlations of the predicted and recorded electric field than literature conductivities, albeit without significantly different slopes. They further incorporated anisotropic conductivities from diffusion-weighted imaging, but the inclusion of anisotropy did not improve the accuracy of the distribution of electric fields in the brain (Huang et al., 2017). The effect of anisotropic conductivities may play a larger role when modeling neuronal effects. Deep brain stimulation simulations have shown that anisotropic conductivities result in asymmetric voltage spread, thus altering the distribution of axonal activation (Butson et al., 2007; Chaturvedi et al., 2010). Furthermore, a subdural cortical stimulation study of pyramidal neurons showed that anisotropy altered the spatial extent of excitation thresholds (Seo et al., 2015). Finally, the electrode-tissue interface impedance is a critical parameter for modeling electrophysiological responses to deep brain stimulation (Butson et al., 2006) and chronic subdural DECS to treat epilepsy (Sillay et al., 2013). Further investigation of the effects of higher-order conductivity parameters will better inform their relevance in models of therapeutic applications of DECS.

We observed greater errors in our predicted voltages near the stimulating electrodes where the voltages were higher. We expect the error to drop off with distance from the stimulus. However, for therapeutic applications, we are most interested in regions nearest the stimulating electrodes where neuromodulation is likely to occur. Although we sampled voltages near and distant to the stimulating electrodes, electrodes that recorded greater voltages drove the correlation values shown in Figure 7A, most notable for patients 2, 5, and 6 whose correlation values were lower. These electrodes also influenced the slopes of our predictive models. Validation of electric field models with intracranial recordings is currently the gold standard; nonetheless, noise in the recording values has been shown to effect slope estimates and may have introduced uncertainty in our predictive models (Puonti et al., 2020). Noisy recording values are more likely for electrodes recording lesser voltages, approaching zero, where the signal-to-noise ratio is less. Slope estimates may be improved by systematically identifying a specific distance or voltage threshold at which data points should be excluded. We chose to objectively sample all data and investigate the effects of all possible contacts. Although the present study incorporated both grid and strip electrodes experimentally and in the models, we recorded only from the grid electrodes; unanswered questions remain regarding the effects of brain shift and CSF depth on strip electrodes. Alternative explanations of the greater errors near the stimulating electrodes may be due to a combination of the modeling limitations mentioned above, such as poor characterization of the electrode-tissue interface, conductivity, or electrode localization. Additional parameters to consider may be mesh resolution near the electrodes or finite element interpolation errors (Howell et al., 2014). For some future DECS model applications (e.g., estimating neural activation), altered or even adaptive mesh resolutions near the stimulating electrodes are warranted.

Finally, FEM models of DECS could be improved by alternative methods to minimize the electrode localization uncertainty after post-implantation brain shift. One such method could model cortical anatomy from postoperative imaging instead of co-registering electrode locations to the pre-operative anatomy. This approach would place the electrodes and brain anatomy in a single neuroanatomical space and eliminate the need for electrode projection. Intra-operative photos may also decrease the uncertainty of electrode localization but require a more time-consuming process to identify all electrode locations and may not be available at all clinical sites investigating DECS (Pieters et al., 2013). However, for both postoperative and intraoperative imaging, the effect of cortical anatomy shift throughout the duration of implantation remains unknown, and the relevance of this shift to ECoG interpretation (LaViolette et al., 2011).

### Clinical Impact

The DECS models used in this study assessed the accuracy of the predicted voltages recorded at the surface of the brain. Although the therapeutic mechanisms of DECS remain unclear, the voltages predicted with these models could be coupled with models that predict the patient-specific neuronal response to stimulation, such as volume of tissue activated models and fiber activation models (Butson et al., 2007; Gunalan et al., 2017). These models have been used to advance the field of deep brain stimulation for numerous disorders by identifying predictors of therapeutic response based on the local target stimulated (Butson et al., 2011; Dembek et al., 2019; Elias et al., 2020), the specific fiber pathways stimulated (Howell et al., 2019), and the connectivity of local stimulated regions to distant targets (Horn et al., 2017; Johnson et al., 2020). Combining these methods with our proposed model may provide insights into the therapeutic mechanisms of cortical responsive neurostimulation for epilepsy. Furthermore, these models may serve as a foundation to explore closed-loop stimulation for other disorders, like Parkinson’s disease, that require cortical electrodes for sensing and/or stimulation.

## Conclusion

As the clinical use of DECS continues to grow for applications in epilepsy, stroke, and other neurological disorders, computational models may provide critical insights into the electrophysiological and clinical response. However, there has been limited validation of these models addressing key concerns related to cortical electrode localization. We report a validation study of patient-specific FEM models of subdural DECS in humans that compared the effects of three projection methods and CSF depths on predicted electrode voltages. Of the three electrode projection methods we analyzed, we recommend localizing electrodes using the Hermes projection method to account for brain shift. We additionally suggest modeling a depth of 0 mm of CSF below the electrode. Future models should follow these recommendations to minimize the error of predicted voltages in the brain. Moving forward, these models could be used to investigate the patient-specific neural response to DECS and guide therapy for epilepsy, stroke rehabilitation, and future applications of closed-loop stimulation for many neurological disorders.

## Data Availability Statement

The datasets presented in this study can be found in online repositories. The names of the repository/repositories and accession number(s) can be found below: The code to extract the quasi-static voltages used for validation is available at https://github.com/davidjuliancaldwell/ElectrodeModeling. The code to build the patient-specific models, as well as the data and code to recreate the figures in this manuscript, are available at https://github.com/ChantelC/ECOG_FEM.

## Ethics Statement

The studies involving human participants were reviewed and approved by University of Washington Institutional Review Board. The patients/participants provided their written informed consent to participate in this study.

## Author Contributions

CC: conceptualization, methodology, software, formal analysis, writing—original draft, and visualization. DC: software, formal analysis, investigation, and writing—review and editing. SR and DB: conceptualization and writing—review and editing. AJ: methodology, software, and writing—review and editing. JO and RM: writing—review and editing and funding acquisition. CB: supervision, resources, and writing—review and editing. AD: conceptualization, supervision, and writing—review and editing. All authors contributed to the article and approved the submitted version.

## Conflict of Interest

CB has served as a consultant for NeuroPace, NeuraModix, Advanced Bionics, Boston Scientific, Intelect Medical, Abbott, and Functional Neuromodulation and he holds intellectual property related to neuromodulation therapy. The remaining authors declare that the research was conducted in the absence of any commercial or financial relationships that could be construed as a potential conflict of interest.

## Publisher’s Note

All claims expressed in this article are solely those of the authors and do not necessarily represent those of their affiliated organizations, or those of the publisher, the editors and the reviewers. Any product that may be evaluated in this article, or claim that may be made by its manufacturer, is not guaranteed or endorsed by the publisher.

## Footnotes

1
https://www.slicer.org/

2
www.seg3d.org

3
www.scirun.org
