# Supplementary material for: Validating Patient-Specific Finite Element Models of Direct Electrocortical Stimulation
Source: Front Neurosci. 2021 Aug 2;15:691701. doi: 10.3389/fnins.2021.691701 (PMC8365306; doi:10.3389/fnins.2021.691701)
Supplement: Supplementary file 1 [file Image_1.pdf]

Supplementary Material

1 Supplementary Figures

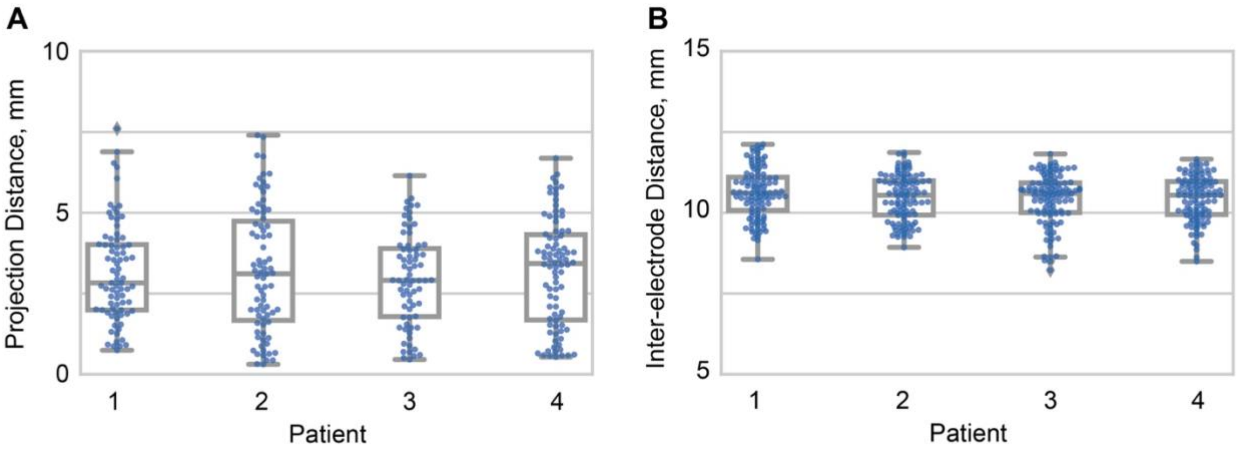

Supplementary Figure 1. Dykstra projection and inter-electrode distances. (A) Boxplots of the projection distance across electrodes within a patient. Patients are ordered from least to greatest median Hermes projection distance. (**B**) Boxplots of the within-patient inter-electrode distances. Each data point represents a pair of neighboring electrodes on the ECoG grid. Data are not shown for patients 5 and 6 for whom the Dykstra projection method did not converge.

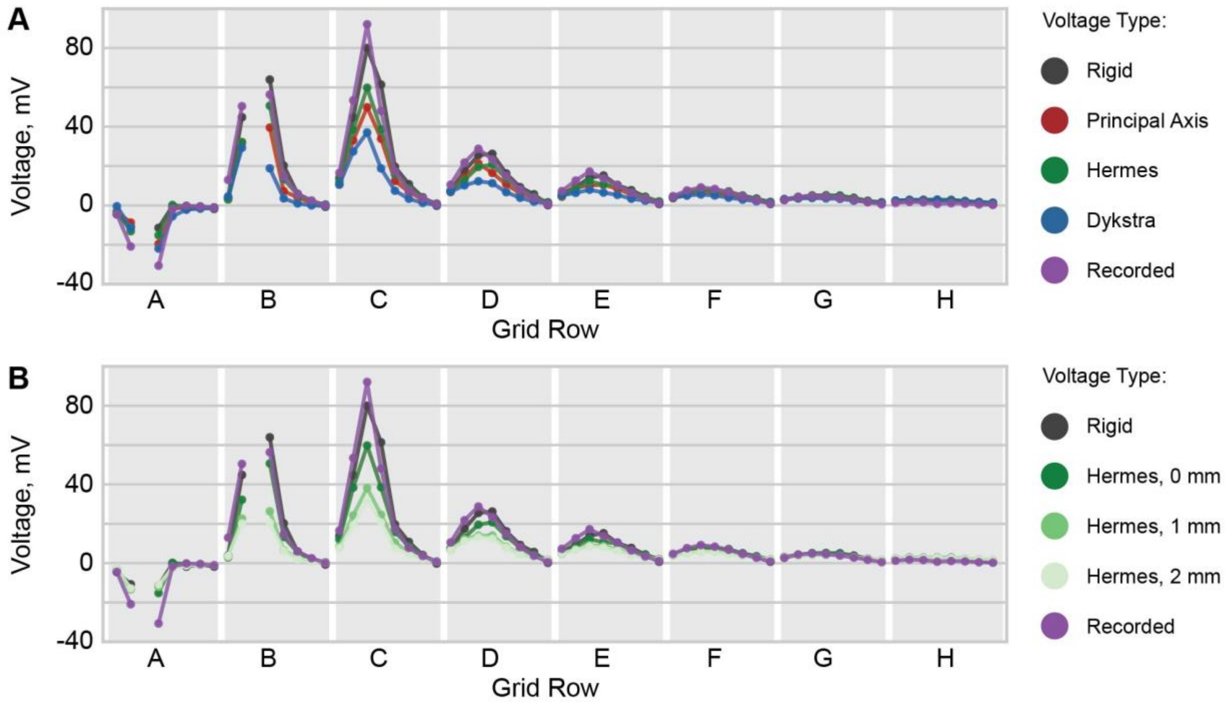

Supplementary Figure 2. (A) Predicted voltage for all projection methods at 0 mm CSF, as well as the rigid model (grey) and recorded voltage (purple). Each alphabetized separation is a horizontal strip

of electrodes on the ECoG grid (labels shown in Fig. 5B). (**B**) Predicted voltages for the Hermes method at 0, 1, and 2 mm of CSF.
